# *In Vitro* Evaluation of the Impact of the Probiotic *E. coli* Nissle 1917 on *Campylobacter jejuni*’s Invasion and Intracellular Survival in Human Colonic Cells

**DOI:** 10.3389/fmicb.2017.01588

**Published:** 2017-08-22

**Authors:** Yosra A. Helmy, Issmat I. Kassem, Anand Kumar, Gireesh Rajashekara

**Affiliations:** ^1^Food Animal Health Research Program, Department of Veterinary Preventive Medicine, Ohio Agricultural Research and Development Center, The Ohio State University, Wooster OH, United States; ^2^Department of Animal Hygiene, Zoonoses and Animal Ethology, Faculty of Veterinary Medicine, Suez Canal University Ismailia, Egypt; ^3^Department of Nutrition and Food Sciences, Faculty of Agricultural and Food Sciences, American University of Beirut Beirut, Lebanon

**Keywords:** probiotic, *Campylobacter*, *E. coli* Nissle 1917, HT-29 cells, adhesion, invasion, intracellular survival, tight junctions

## Abstract

*Campylobacter jejuni* is a leading cause of bacterial food poisoning in humans. Due to the rise in antibiotic-resistant *Campylobacter*, there exists a need to develop antibiotic-independent interventions to control infections in humans. Here, we evaluated the impact of *Escherichia coli* Nissle 1917 (EcN), a probiotic strain, on *C. jejuni’s* invasion and intracellular survival in polarized human colonic cells (HT-29). To further understand how EcN mediates its impact, the expression of 84 genes associated with tight junctions and cell adhesion was profiled in HT-29 cells after treatment with EcN and challenge with *C. jejuni*. The pre-treatment of polarized HT-29 cells with EcN for 4 h showed a significant effect on *C. jejuni*’s invasion (∼2 log reduction) of the colonic cells. Furthermore, no intracellular *C. jejuni* were recovered from EcN pre-treated HT-29 cells at 24 h post-infection. Other probiotic strains tested had no significant impact on *C. jejuni* invasion and intracellular survival. *C. jejuni* decreased the expression of genes associated with epithelial cells permeability and barrier function in untreated HT-29 cells. However, EcN positively affected the expression of genes that are involved in enhanced intestinal barrier function, decreased cell permeability, and increased tight junction integrity. The results suggest that EcN impedes *C. jejuni* invasion and subsequent intracellular survival by affecting HT-29 cells barrier function and tight junction integrity. We conclude that EcN might be a viable alternative for controlling *C. jejuni* infections.

## Introduction

*Campylobacter* *jejuni* is a leading cause of foodborne gastroenteritis worldwide (Fullerton et al., 2007; CDC, 2013; Kaakoush et al., 2015). *Campylobacter* is also associated with post-infectious neurological and joint disorders, such as the Guillain-Barre’ syndrome, the Miller Fisher syndrome, and reactive arthritis (Nachamkin et al., 1998; Zia et al., 2003; CDC, 2014; Mohan, 2015; Kassem et al., 2016b). In the majority of the cases, *Campylobacter* infections are self-limiting and treatment mainly relies on supportive therapy, while antibiotics can be deployed in severe cases (Alemka et al., 2010). However, the emergence of antibiotic-resistant *Campylobacter* strains has emphasized a need to develop alternatives to treat human infections (Kassem et al., 2016a). Of particular interest is exploiting probiotic bacteria as an antibiotic-independent approach to enhance the hosts’ immunity and control infections. The application of probiotic bacteria has also received wide attention as a potential intervention to limit the use of antibiotics in food animal production. The latter is proposed to significantly reduce the impact of agricultural practices on the emergence of antibiotic-resistant foodborne pathogens that affect public health (Sahin et al., 2015).

Probiotic bacteria are defined as non-pathogenic and viable microorganisms that can confer beneficial effects on the host by maintaining gut microbial balance and homeostasis and facilitating mucosal repair in the gastrointestinal tract (Behnsen et al., 2013; Mohan, 2015). Additionally, probiotic bacteria have been shown to attenuate the impact of several enteropathogens (Corry et al., 1995; Sherman et al., 2005). For example, *Escherichia coli* Nissle 1917 (EcN) reduced the invasion of human intestinal epithelial cells by important bacterial pathogens, including *Salmonella enterica* serovar Typhimurium, *Shigella flexneri*, enteroinvasive *E. coli*, *Listeria monocytogenes*, and *Yersinia enterocolitica* (Altenhoefer et al., 2004; Kleta et al., 2006; Reissbrodt et al., 2009; Sonnenborn and Schulze, 2009). EcN is one of the widely characterized probiotic strains that demonstrate beneficial activity in both humans and animals (Kruis et al., 2004; Schultz et al., 2004; von Buenau et al., 2005; Krammer et al., 2006; Schroeder et al., 2006; Henker et al., 2008). EcN persistently colonizes its hosts (Schultz, 2008) and has been shown to (1) produce antimicrobial compounds such as bacteriocins or microcins, (2) modulate host immune responses, and (3) participate in competitive exclusion of pathogens (Helwig et al., 2006; Ukena et al., 2007; Zyrek et al., 2007; Kamada et al., 2008; Bar et al., 2009; Bickert et al., 2009; Reissbrodt et al., 2009; Trebichavsky et al., 2010; Behnsen et al., 2013). Furthermore, EcN interacts with intestinal epithelial cells to express proteins that mediate normal gut barrier functions, normalize gut permeability, and improve mucosal integrity (Zyrek et al., 2007). Consequently, EcN has a plethora of desirable probiotic properties, which can be beneficial to the overall gut health and provide protection against enteric infections.

In contrast to some other probiotic strains (Wine et al., 2009), the impact of EcN on *C. jejuni*’s interaction with intestinal cells has not been characterized. Consequently, we investigated the effect of EcN on *C. jejuni*’s invasion of human intestinal epithelial cells *in vitro*. Furthermore, we assessed the response of intestinal cells to *C. jejuni* in the presence and absence of EcN using human tight junction RT^2^ Profiler PCR Arrays (Qiagen, Array # PAHS-143Z), which evaluates the expression of 84 genes associated with tight junctions (Moradipoor et al., 2016). The integrity of cell to cell junctions (including tight junctions) is key to normal gut barrier functions and permeability, which affect the pathophysiology of enteric infections (Bischoff et al., 2014). This is very important, because *C. jejuni* was shown to impact tight junctions in intestinal epithelial monolayers, causing the redistribution of occludin (a tight junction transmembrane protein) from an intercellular to an intracellular location and potentially compromising the intestinal barrier (Chen et al., 2006). Therefore, we also used a polarized human colon cells (HT-29), which was shown to be valuable for evaluating the impact of pathogens on cell barrier permeability, transcytosis mechanisms, and cell invasion (Bras and Ketley, 1999). Furthermore, the HT-29 cells have been considered as one of the more appropriate cell types for assessing *Campylobacter* virulence *in vitro* (Haddad et al., 2010).

## Materials and Methods

### Bacterial Strains and Growth Conditions

*Campylobacter jejuni* 81-176 is a well-characterized invasive and wild type strain that has been routinely used as a “global model” in studies that characterize *C. jejuni* virulence and host pathogen interactions (Korlath et al., 1985; Hendrixson and DiRita, 2004; Papp-Szabo et al., 2005; Hofreuter et al., 2006). In this study, *C. jejuni* 81-176 was routinely cultured using Mueller-Hinton (MH) agar (Difco) with a *Campylobacter* selective supplement (CSS) (SR0117; Oxoid) at 42°C under microaerobic conditions (5% O_2_, 10% CO_2_, and 85% N_2_) (Kassem et al., 2012). *E. coli* strain Nissle 1917 (EcN) was cultured aerobically using Luria-Bertani (LB) broth at 37°C to achieve logarithmic growth. Other bacterial strains, *Lactobacillus rhamnosus* GG (LGG; ATCC 53703), *Lactobacillus acidophilus* NCFM (LA; ATCC 700396), and *Bifidobacterium animalis* subsp. *Lactis* (Bb-12; Christian Hansen, Ltd, Hørsholm, Denmark) were cultured using MRS (de Man, Rogosa and Sharpe) media under anaerobic condition, which was generated using the GasPak^TM^ EZ Anaerobe Container System Sachets (BD, United States) (Kassem et al., 2012). To facilitate the growth of Bb-12, the MRS broth was supplemented with 0.05% cysteine hydrochloride. LA, LGG, and Bb-12 were grown at 37°C for 18 h (Kumar et al., 2014).

### The Impact of Different Probiotic Strains on Adhesion, Invasion, and Intracellular Survival of *C. jejuni* in HT-29 Cells

HT-29 (Human Colorectal Adenocarcinoma Cell Line; ATCC HTB-38) cells were maintained in complete Dulbecco’s modified Eagle’s medium (DMEM, Gibco) supplemented with 10% fetal bovine serum (FBS, Gibco), 2 mM L-glutamine, 5 mM galactose, 1% antibiotic and 0.1 mM non-essential amino acids (Altenhoefer et al., 2004). The cells were incubated at 37°C in a humidified atmosphere with 5% CO_2_. Prior to each experiment, polarized cells were prepared after seeding 1.4 × 10^5^ HT-29 cells into each well of a 96-well cell culture plate, which was then incubated for 3–4 days (Alemka et al., 2010). The polarized HT-29 cells were washed with- and incubated in DMEM containing no antibiotics and FBS prior to challenge with bacteria (Otte and Podolsky, 2004).

To evaluate the effect of probiotic bacteria on *C. jejuni*’s adhesion to HT-29 cells, the probiotic bacteria (EcN, LGG, LA, Bb-12) were grown to early exponential phase, pelleted, washed two times with Dulbecco’s phosphate-buffered saline (DPBS), and re-suspended in DMEM. Hundred microliter of each suspension (1 × 10^7^ CFUs) was added to the wells containing the HT-29 monolayers, which were then incubated for 4 h (Altenhoefer et al., 2004). The HT-29 cells were then washed three times and infected with 1.7 × 10^7^ CFUs of *C. jejuni* 2 h. After this, the infected HT-29 cells were washed three times with DBPS and the adherent *C. jejuni* CFUs were enumerated after lysis with 0.1% Triton X-100, serial dilution (10-fold), and spreading onto MH agar plates containing CSS.

To determine the effect of probiotics on *C. jejuni*’s invasion of HT-29 cells, the HT-29 cells were pre-treated with the different probiotic bacteria and *C. jejuni* as described above. However, following the 2 h incubation with *C. jejuni*, the HT-29 cells were washed three times with DPBS and treated with DMEM containing 150 μg ml^-1^ gentamicin for an additional 1 h. The HT-29 cells were then washed twice with DPBS, lysed with 0.1% Triton X-100 and *C. jejuni* CFUs were quantified as described above.

To assess *C. jejuni*’s intracellular survival, the HT-29 cells were treated as described above. However, after the gentamicin treatment, the HT-29 cells were washed and incubated again for 24 h in fresh DMEM containing 10 μg/ml^-1^ gentamicin (Altenhoefer et al., 2004; Schierack et al., 2011; Kassem et al., 2012). After this, the HT-29 cells were washed twice and lysed to quantify *Campylobacter* CFUs as described above. These experiments were repeated on two separate occasions and each sample was replicated four times per experiment.

We also evaluated the effect of different incubation time of EcN with HT-29 cells on *C. jejuni*’s adhesion, invasion, and intracellular survival. For this purpose, polarized HT-29 cells were incubated with EcN (1.7 × 10^7^ CFUs) for 1, 2, 3, and 4 h before infection with *C. jejuni* as described above.

### Assessment of the Impact of EcN’s Heat Killed Cells and Cell-Free Supernatant on *C. jejuni’s* Interaction with HT-29 Cells

Possible interactions between EcN and *C. jejuni* were evaluated further as follows:

(A) Treatment of HT-29 cells with heat-killed EcN:

Heat- killed EcN were prepared by heating exponentially grown cultures to 65°C for 5 min and the loss of EcN viability was confirmed by spreading the cultures on LB agar. The polarized HT-29 cells were then incubated for 4 h with heat-killed EcN (equivalent to ∼1.7 × 10^7^ CFUs) before infection with *C. jejuni* as described above. Additionally, heat-killed EcN and *C. jejuni* mixtures (1:1 and 10:1 v/v) were pre-incubated at room temperature for 0, 1, 2, 3, and 4 h and then used to infect the polarized HT-29 cells.

(B) Treatment of HT-29 cells with EcN cell-free supernatant:

EcN-free supernatant was prepared from exponentially grown cultures, which were centrifuged at 5000 × *g* for 10 min. The supernatant was then collected and filtered through a sterile membrane (0.2 μm pore size) (Corning, Germany) and confirmed to be EcN free by culturing on LB agar. The EcN-free supernatant (prepared from cultures containing the equivalent of 1.7 × 10^7^ CFUs) was used to treat the HT-29 cells prior to *C. jejuni* infection as described above. Additionally, cell-free supernatants were prepared from EcN cultures containing the equivalent of 1 and 10X the number of *C. jejuni* CFUs. These supernatants were also used to suspend *C. jejuni* at room temperature for 0, 1, 2, 3, and 4 h prior to infection.

In each of the experiments above, *C. jejuni’*s adherence, invasion, and intracellular survival were assessed by determining the number of *C. jejuni* CFUs ml^-1^ as described earlier. All experiments were repeated at least two times using four replicates of each sample per experiment.

### Human Tight Junctions RT^2^ Profiler PCR Arrays Analysis

The expression of 84 tight junction-associated genes was determined using the human tight junctions RT^2^ Profiler PCR Arrays (Qiagen, Array # PAHS-143Z) (Moradipoor et al., 2016). Subsequently, polarized HT-29 cells were treated with EcN for 4 h and infected with *C. jejuni* for 2 h (invasion) and 24 h (intracellular survival). Untreated and unchallenged HT-29 cells and those that were challenged with EcN and *C. jejuni* were used as controls, respectively. Total RNA was extracted from the HT-29 cells using the TRIzol reagent (Life Technologies, United States) and the miRNeasy Mini Kit (Qiagen) and purified of DNA traces as described by the manufacturer (Qiagen). RNA quality and quantity were determined using nanodrop 2000 C spectrophotometer (Thermoscientific) and by electrophoresis in agarose gels.

Approximately, 5 μg of purified RNA were used to synthesize cDNA using the Qiagen RT^2^ First Strand Kit (Qiagen). As specified by the manufacturer, cDNA was added to the RT^2^ SYBR Green qPCR Master Mix (Qiagen) and 25 μl of the mixture were added to each well of a 96 well plate that pre-contained gene-specific primer sets (RT^2^ Profiler PCR Arrays) as described by the manufacturer. qRT PCR was performed using a Mastercycler^®^ RealPlex^2^ (Eppendorf). The threshold cycle (Ct) values were calculated for each gene and normalized using the house-keeping genes included in the Arrays. Fold-changes in gene expression (between treated and control samples) were calculated using the ΔΔCt method (Cao et al., 2015). Significantly, affected genes were then analyzed using the Ingenuity Pathway Analysis (IPA) software^1^ (Kumar et al., 2014) to identify potential functions and cellular pathways that were modulated in HT-29 cells in response to EcN and *C. jejuni*.

### Statistical Analysis

Data generated from the gentamicin protection assays (adherence, invasion, and intracellular survival) were presented as means ± standard deviations. ANOVA followed by the Tukey test was used to analyze these data and a *P*-value < 0.05 was used to determine statistically significant differences between means. A fold change of ±1.5 ≥ or ≤ 1.5 and a *P*-value ≤ 0.05 were used to determine statistically significant differences in gene expression.

## Results

### The Effect of Probiotic Bacteria on the Interaction of *C. jejuni* with HT-29 Cells

**FIGURE 1:**
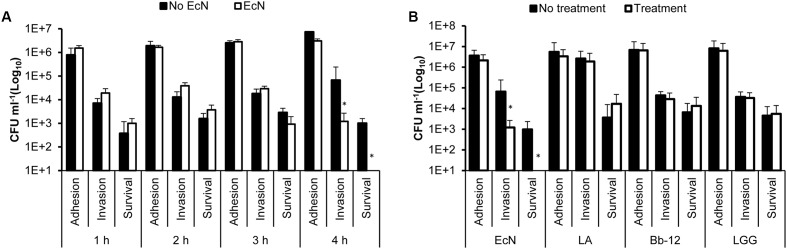
*Campylobacter jejuni*’s adhesion to, invasion of and intracellular survival in HT-29 cells pretreated with **(A)** EcN for 1, 2, 3, and 4 h; **(B)** EcN, *Lactobacillus acidophilus* NCFM (LA), *Bifidobacterium animalis* subsp. *Lactis* (Bb-12) and *Lactobacillus rhamnosus* GG (LGG) for 4 h. ^∗^Indicates statistically significant differences (*P* < 0.05) in *C. jejuni* CFU numbers between probiotic-treated and non-treated HT-29 cells. The experiments were repeated at least two times and samples were processed in four replicates in each experiment.

To determine the impact of EcN on the interaction of *C. jejuni* with polarized HT-29 cells, we incubated EcN with the HT-29 cells for 1, 2, 3, and 4 h prior to infection with *C. jejuni.* The treatment with EcN for 4 h resulted in the significant reduction in *C. jejuni’s* invasion and intracellular survival (**Figure 1A**). To compare the effect of EcN to other commonly known probiotic bacteria, we treated the polarized HT-29 cells with EcN, LA, LGG, and Bb-12 for 4 h then infected these cells with *C. jejuni* 81-176, respectively. Our results show that LA, LGG, and Bb-12 did not significantly impact the interaction of *C. jejuni* with the HT-29 cells. However, while EcN did not significantly impact the adherence of *C. jejuni* to HT-29 cells, EcN significantly reduced (*P* < 0.05) *C. jejuni’s* invasion by ∼2 logs CFU ml^-1^ in comparison to the control (HT-29 cells not treated with EcN). Furthermore, no intracellular *C. jejuni* were recovered from EcN-treated HT-29 cells in comparison to the control (**Figures 1A,B**).

### The Impact of EcN Cell Free Supernatant and Heat-Killed Cells on the Interaction of *C. jejuni* with HT-29 Cells

**FIGURE 2:**
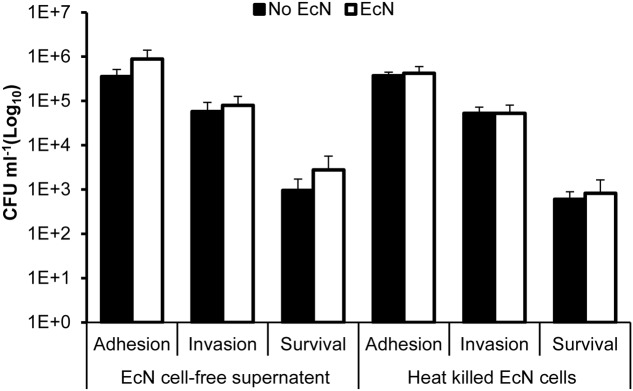
*Campylobacter jejuni*’s adhesion to, invasion of and intracellular survival in HT-29 cells pretreated with EcN free supernatant and heat killed cells for 4 h. Heat-killed EcN were generated by heating exponentially growing cultures to 65°C for 5 min and checking the loss of viability by culturing on LB agar plates. EcN-free supernatant was prepared from exponentially growing cultures, which were centrifuged and filtered through a sterile membrane. The experiments were repeated at least two times and samples were processed in four replicates in each experiment.

**FIGURE 3:**
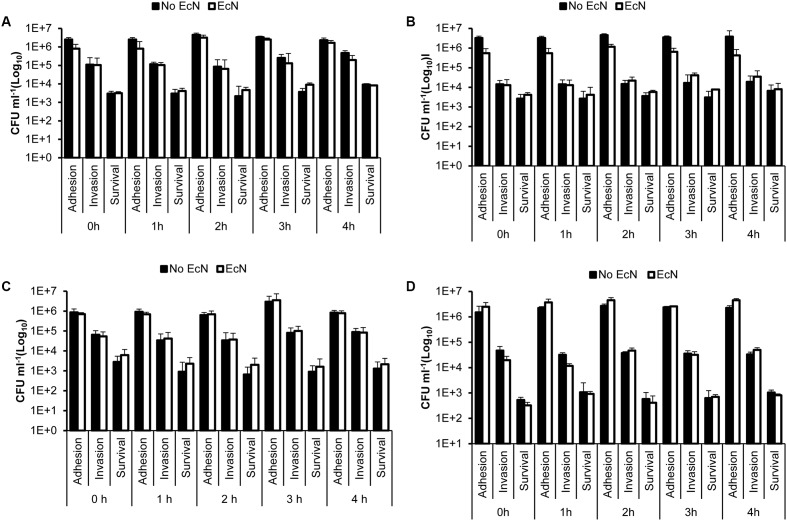
*Campylobacter jejuni*’s adhesion to, invasion of and intracellular survival in HT-29 cells following co-incubation of *C. jejuni* with EcN free supernatant and heat killed cells prior to infection of HT-29 cells. **(A)** Heat-killed EcN and *C. jejuni* 81-176 were co-incubated (1:1) for 0, 1, 2, 3, and 4 h at room temperature before the HT-29 cells were infected with the mixture for 2 h. **(B)** Heat-killed EcN and *C. jejuni* 81-176 were co-incubated (10:1) for 0, 1, 2, 3, and 4 h at room temperature before the HT-29 cells were infected with the mixture for 2 h. **(C)** EcN-cell free supernatant and *C. jejuni* 81-176 were co-incubated for 0, 1, 2, 3, and 4 h at room temperature before the HT-29 cells were infected with the mixture for 2 h. The cell-free supernatant was prepared from cultures containing the same number of EcN CFUs as *C. jejuni* (1X). **(D)** EcN-cell free supernatant and *C. jejuni* 81-176 were co-incubated for 0, 1, 2, 3, and 4 h at room temperature before the HT-29 cells were infected with the mixture for 2 h. The cell-free supernatant was prepared from cultures containing 10X more EcN CFUs than *C. jejuni* (10X). The experiments were repeated at least two times and samples were processed in four replicates in each experiment.

The pretreatment of HT-29 cells with EcN cell-free supernatant and heat-killed EcN cells for 4 h prior to infection with *C. jejuni* showed no significant impact (*P* > 0.05) on *C. jejuni’s* interaction with HT-29 cells (**Figure 2**). Additionally, pre-incubation of C. *jejuni* at room temperature with EcN cell-free supernatants and with heat killed EcN cells for 0, 1, 2, 3, and 4 h prior to infection of HT-29 cells also showed no significant impact on *C. jejuni’s* interaction with HT-29 cells (**Figures 3A–D**). Direct spreading of aliquots form the pre-incubated mixtures on MH agar plates showed no change in *C. jejuni* CFUs, which further confirmed the insignificant impact of EcN’s cell-free supernatants and heat killed cells on *C. jejuni* viability (data not shown).

### The Impact of EcN on the Expression of Tight Junction-Associated Genes in HT-29 Cells

RT^2^ Profiler PCR Arrays were used to evaluate the impact of EcN on tight junctions-associated gene expression in HT-29 cells. Collectively, the three treatments (EcN, *C. jejuni*, and EcN + *C. jejuni*) affected 76 out of 84 genes included in the arrays (Supplementary Table S1). The detailed description of differentially expressed genes with the fold change values under different treatments is included in Supplementary Table S2. IPA analysis identified two canonical pathways that were significantly modulated by EcN treatment and *C. jejuni* infection. The two major affected canonical pathways were (1) tight junctions and other cell–cell junction signaling (TCS) and (2) cell adhesion and extravasation signaling (CAS).

#### Impact on TCS Associated Genes

**FIGURE 4:**
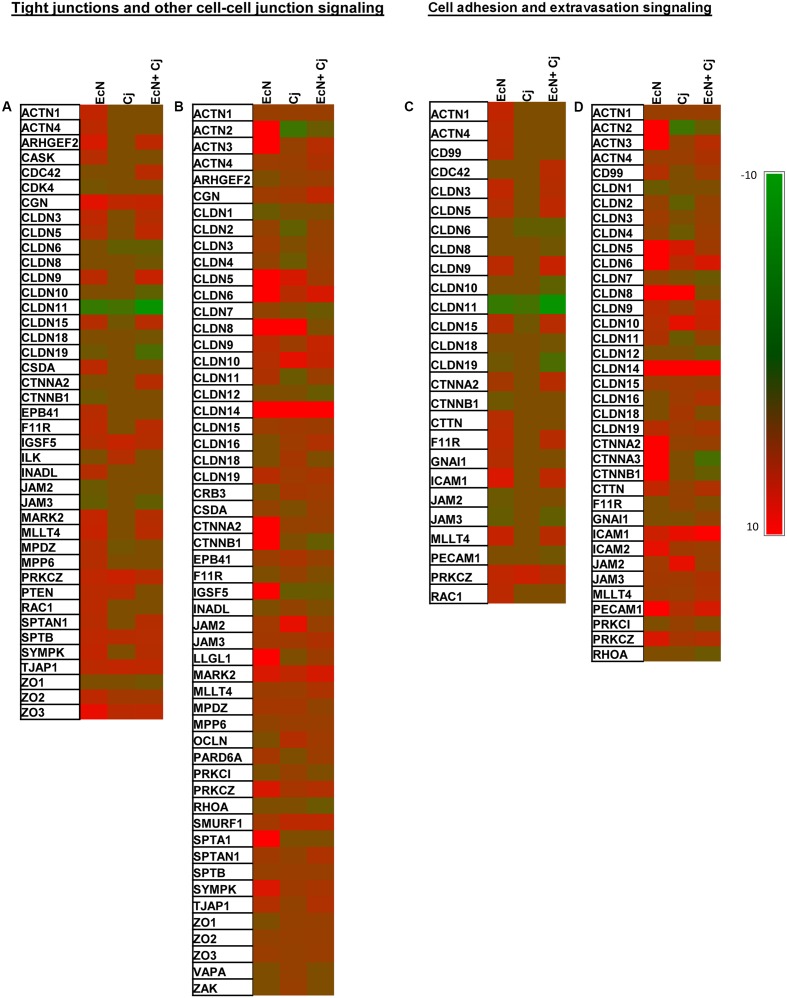
Expression of tight junction associated genes in HT-29 cells with and without EcN and *C. jejuni*. Expression of genes associated with two significantly modulated canonical pathways (1) tight junction and other cell–cell junction signaling (TCS) at 2 h **(A)** and 24 h **(B)** *C. jejuni* post-infection and (2) cell adhesion and extravasation signaling (CAS) at 2 h **(C)** and 24 h **(D)** *C. jejuni* post-infection. The human tight junction RT^2^ Profiler PCR arrays were used to assess the expression of 84 genes in EcN-pretreated cells at 2 and 24 h post-*C. jejuni* infection. Significant change in gene expression was determined by comparison to untreated cells (no EcN and no *C. jejuni*). A fold change of ±1.5 ≥ or ≤ 1.5 and a *P* ≤ 0.05 were used to determine significant differences in gene expression.

At 2 h post-infection with *C. jejuni* (invasion), EcN treatment alone caused significant alteration in the expression of 33 genes (27 up-regulated and 6 down-regulated) in TCS (**Figure 4A**). Interestingly, nine up-regulated genes and three down-regulated genes (encoding CDK4, CTNNB1, and JAM2) were uniquely affected by treatment with EcN alone. *C. jejuni* infection affected the expression of 12 genes (eight up-regulated and four down-regulated), one gene encoding MPDZ (an up-regulated junction associated protein) was uniquely associated with *C. jejuni* (**Figure 4A**). EcN + *C. jejuni* affected 26 genes (18 up-regulated and 8 down-regulated), 8 of which were uniquely associated with this treatment. Notably, in EcN + *C. jejuni*, the expression of the CLDN15 encoding gene was up-regulated in comparison to infection with *C. jejuni* (**Figure 4A**).

After 24 h post-infection with *C. jejuni* (intracellular survival), the expression of 40 genes was up-regulated in HT-29 treated with EcN, and one of these genes (encoding SPTA1) was uniquely associated with EcN (**Figure 4B**). Only the expression of one gene (encoding CLDN1) was down-regulated by EcN. *C. jejuni* infection altered the expression of 44 genes (39 up-regulated and 5 down-regulated). Notably, the down-regulated genes (encoding CLDN2, CLDN4, CLDN11, ACTN2, and IGSF5) associated with the *C. jejuni* treatment were up-regulated in the presence of EcN alone (**Figure 4B**). Additionally, six up-regulated genes (encoding ZAK, F11R, PRKC1, INDAL, VAPA, and CLDN18) were uniquely associated with *C. jejuni* infection. EcN + *C. jejuni* affected the expression of 45 genes (39 up-regulated and 6 down-regulated) (**Figure 4B**). The expression of two genes was uniquely impacted in the EcN + *C. jejuni* treatment and included genes that encoded CLDN12 (claudin), and RHOA (G-protein signaling). In addition, in EcN + *C. jejuni*, the expression of the CLDN2, CLDN4, and CLDN11 encoding genes was down-regulated in comparison to infection with *C. jejuni* (**Figure 4B**).

#### Impact on CAS Associated Genes

At 2 h post-infection with *C. jejuni* (invasion), EcN treatment alone caused significant alteration in the expression of 19 genes (14 up-regulated and 5 down-regulated) associated with CAS (**Figure 4C**). Of these, six up-regulated genes and two down-regulated genes were uniquely observed in treatment with EcN alone. *C. jejuni* infection affected four genes (one up-regulated and three down-regulated). Notably, one of the down-regulated genes (encoding the claudin, CLND15) was up-regulated in the EcN + *C. jejuni* treatment (**Figure 4C**). Additionally, the expression of 18 genes (10 up-regulated and 8 down-regulated) was affected in EcN + *C. jejuni* and change in the expression of 6 (two up-regulated and four down-regulated) of these genes was uniquely associated with this treatment. The latter genes encoded CDC42, CLDN8, CLDN10, CLDN18, CTNNA2, and PECAM1 (**Figure 4C**).

After 24 h post-infection with *C. jejuni* (intracellular survival), the expression of 29 genes was up-regulated in HT-29 treated cells with EcN, while one gene encoding CLDN1 was down-regulated (**Figure 4D**). Changes in one gene (encoding CLDN1) were uniquely observed in the EcN treatment (**Figure 4D**). In comparison to untreated controls, EcN treatment caused the up-regulation of genes encoding ACTN2, and ACTN3 by 42.5, and 87 folds, respectively (**Figure 4D**). *C. jejuni* infection altered the expression of 28 genes (24 up-regulated and 4 down-regulated), and the impact on expression of three of the up-regulated genes (encoding CLDN18, F11R, and PRKC1) was only observed in this treatment (**Figure 4D**). EcN + *C. jejuni* affected the expression of 32 genes (26 up-regulated and 6 down-regulated) and the expression of two of the down-regulated genes (encoding CLDN12, and RHOA) was uniquely associated with this treatment. In addition, in EcN + *C. jejuni*, the expression of the CLDN2, CLDN4, and CLDN11 encoding genes was up-regulated in comparison to infection with *C. jejuni* (**Figure 4D**).

## Discussion

In light of the increase in antibiotic resistant *C. jejuni* (Kassem et al., 2016a), there is a need to proactively devise alternative approaches to control the proliferation of this pathogen and cognate infections. Several studies have recently investigated the effect of probiotic bacteria on *C. jejuni* infections in cell lines (*in vitro*) and in animal models such as chickens, a primary host and source of this bacterium (Baffoni et al., 2017; Ekmekciu et al., 2017; Johnson et al., 2017). *Lactobacillus*, *Bacillus*, and *Enterococcus* have been among the most commonly researched probiotic bacteria against *C. jejuni* (Johnson et al., 2017). For example, Chaveerach et al. (2004) showed that probiotic bacteria, *Lactobacilli* and *Bifidobacteria*, had a negative effect on the growth of different strains of *C. jejuni* and *C. coli.* However, previous studies also suggest probiotic bacteria did not always significantly affect *C. jejuni* colonization and that the desirable antagonistic impacts appeared to vary according to the probiotic strain. For example, *Lactobacillus helveticus* R0052 reduced *C. jejuni*’s invasion of human epithelial colon cells (T84), while *L. rhamnosus* strain R0011 did not affect the invasion of these cells (Wine et al., 2009). Despite their promise in laboratory trials, orally administered strains such as *Lactobacillus acidophilus*, *Bacillus subtilis*, and *Enterococcus faecium* did not significantly reduce *C. jejuni* in broiler chickens (Thomrongsuwannakij et al., 2016). Taken together, the aforementioned observations highlight a need to thoroughly evaluate different probiotic bacteria/strains to assess (1) their impact on *C. jejuni*, and (2) the mechanisms that govern the desirable antagonistic effect on this pathogen. To address this need, we evaluated the potential use of the probiotic strain, EcN, to control *C. jejuni in vitro*. This was motivated by previous observations that showed negative impact of EcN on the invasion of human intestinal epithelial cells by several important pathogens, including *Salmonella enterica* serovar Typhimurium and *Listeria monocytogenes* (Altenhoefer et al., 2004; Kleta et al., 2006; Reissbrodt et al., 2009; Sonnenborn and Schulze, 2009). Furthermore, the probiotic properties of EcN and its use in the treatment of various diseases of the digestive tract of humans have been strongly established (Altenhoefer et al., 2004).

In our experimental system, we found that only EcN negatively impacted *C. jejuni*’s invasion of and intracellular survival in polarized HT-29 cells (**Figure 1**) and in another human intestinal epithelial cell line (Caco-2) (Supplementary Figure S1). In comparison, the probiotic bacteria strains, LA, Bb-12, and LGG did not significantly affect *C. jejuni*’s interaction with the HT-29 cells (**Figure 1B**). EcN cell-free supernatant and heat-killed EcN did not affect *C. jejuni*’s interaction with intestinal cells (**Figures 2**, **3**). This suggested that the antagonistic impact of EcN (1) might require live EcN to inhibit *C. jejuni*, and (2) might not significantly include metabolites secreted into the culture supernatant. This is not surprising, because it is known that viable probiotic bacteria and their cell-free supernatant differ in their ability to protect against pathogens (Campana et al., 2017). Furthermore, the optimal impact of EcN was observed after incubating this probiotic bacterium with intestinal cells for 4 h before infection with *C. jejuni* (**Figure 1A**). This suggested that the contact time between the viable probiotic bacterium and intestinal cells was crucial. Notably, in a previous report, it was suggested that EcN on the surface of the intestinal cells impedes the ability of potential pathogens to exert their impact (Boudeau et al., 2003). This might be attributed to the ability of live probiotic bacteria to compete with pathogens for nutrients and for binding to intestinal cell receptors. Additionally, we observed that EcN and *C. jejuni* did not significantly co-aggregate in co-cultures (data not shown). Taken together, this suggests that EcN might be forming a physical barrier between the pathogen and intestinal cells and/ or inducing intestinal cell properties that might resist infection. The latter is plausible based on reports that probiotic bacteria can increase tight junction integrity and enhance intestinal barrier function and permeability to resist bacterial invasion (Putaala et al., 2008). Furthermore, this corroborated previous studies that reported that EcN’s probiotic activity might be mediated via enhancing the intestinal barrier through the up-regulation of tight junction-associated proteins (Schulze and Downward, 2001; Ukena et al., 2007).

Based on our analysis above, we evaluated the expression of 84 genes associated with tight- and other cell to cell junctions to further analyze how EcN mediates its impact (**Figure 4**). Notably, several probiotic strains appear to affect the expression of occludins and cingulin (Anderson et al., 2010; Miyauchi et al., 2012; Patel et al., 2012). This is important, because *C. jejuni* infection can increase the epithelial cells permeability and induce epithelial barrier disruption, which in turn might facilitate the invasion of the gut (Wine et al., 2008). Therefore, EcN’s impact on epithelial cell junctions might (1) reduce *C. jejuni*’s impact on epithelial cells and (2) prevent the pathogen from possibly entering via the paracellular pathway to cause further damage to the cells (Ulluwishewa et al., 2011). In our study, EcN differentially impacted the expression of tight junction associated genes in HT-29 cells at 2 h (44 genes) and 24 h (55 genes), including genes encoding occludins (ZO-2 and ZO-3) and spectrins (Supplementary Table S1 and **Figure 4**). In several other studies, EcN increased the expression of ZO-2 and caused the redistribution of this protein, which lead to restoration of a disrupted epithelial barrier (Bischoff et al., 2014), while spectrins have been implicated in the stabilization and remodeling of epithelial junctions (Naydenov and Ivanov, 2011). Furthermore, in our study, EcN affected the expression of genes encoding claudins, including claudin 3, 5, 9, 15 at 2 h and claudin 2–14, 15, and 19 at 24 h (**Figure 4**). Interestingly, claudin 3, 4, 5, and 8 are associated with enhancing tight junctions and reducing space between two neighboring cells and decreasing paracellular permeability (Ulluwishewa et al., 2011). Furthermore, the EcN-associated differential expression of genes encoding actinins, catenins/cadherins, spectrins, and other adhesion molecules (**Figure 4**) might contribute to intestinal homeostasis by affecting adherens junctions, which are cell to cell anchoring structures that contribute to organization of the epithelium (Harris and Tepass, 2010). Taken together, we suggest that the presence of EcN appears to stimulate the expression of genes that that enhance cell to cell junction and intestinal barrier integrity. This “priming” effect might increase the resistance of the HT-29 cells to infection.

In our study, EcN stimulated the expression of genes that were either not affected or were downregulated by *C. jejuni* (**Figure 4** and Supplementary Table S1). For example, at 2 h, the expression of genes encoding claudin 3, 5, and 9 and F11R, SYMPK, ARCHGEF2, ESAM, ICMA1, MARK2, and MLLT4 was only upregulated in the EcN and the EcN + *C. jejuni* treatments. Similarly, at 24 h, genes encoding CD99, CLDN3, CSDA, ESAM, LLGL1, and PARD6A were only upregulated in the EcN and the EcN + *C. jejuni* treatments (**Figure 4** and Supplementary Figure S1). Furthermore, EcN antagonized the impact of *C. jejuni* on the expression of certain genes in the intestinal cells. For example, genes encoding claudin 15 at 2 h and claudin 2, 4, and 11 at 24 h were down-regulated when HT-29 cells were infected with *C. jejuni* only. However, these genes were upregulated when the intestinal cells were pre-treated with EcN (**Figure 4** and Supplementary Figure S1). These genes either directly (structure) or indirectly (signaling) affect cell junctions and adherence (Matter and Balda, 2003), which further suggest that EcN mediates its protective impact against *C. jejuni* by enhancing the ability of the intestinal cells to resist infection. Although it is interesting to explore the specific role of individual genes, it should also be noted that EcN’s beneficial effects should be considered in terms of the overall impact on all of the investigated genes and the associated cellular pathways.

The data presented in this study show that the pretreatment of the intestinal cells with EcN can protect against *C. jejuni*’s invasion and intracellular survival. It is likely that this antagonistic activity is facilitated via the probiotic EcN’s impact on HT-29 cell to cell junctions. Our data support the need for future studies that will test the effect of EcN on *C. jejuni* in cognate animal models such as mice and chickens. This may facilitate development of a potentially effective antibiotic-independent approach to control *C. jejuni* in humans and other animal reservoirs.

## Author Contributions

YH, IK, and GR conceived and designed the study. YH and AK conducted the experiments. YH, IK, and GR analyzed the data and wrote the manuscript. All authors read and approved the final manuscript.

## Conflict of Interest Statement

The authors declare that the research was conducted in the absence of any commercial or financial relationships that could be construed as a potential conflict of interest.

## Footnotes

1 www.ingenuity.com
