# Supplementary material for: In Vitro Evaluation of the Impact of the Probiotic E. coli Nissle 1917 on Campylobacter jejuni’s Invasion and Intracellular Survival in Human Colonic Cells
Source: Front Microbiol. 2017 Aug 22;8:1588. doi: 10.3389/fmicb.2017.01588 (PMC5572226; doi:10.3389/fmicb.2017.01588)
Supplement: Supplementary file 1 [file Table_1.PDF]

Supplemental Table 1: Summary of the tight junction genes expression in response to different treatments. The up-regulated and down-regulated genes were determined using a cutoff ± ≥ 1.5 or ≤ 1.5 and a *P* ≤ 0.05.

	**2h post infection**			**24h post infection**		
**Treatments**	**All genes**	**Up-regulated**	**Down-regulated**	**All genes**	**Up-regulated**	**Down-regulated**
**EcN**	44	37	7	55	54	1
C. jejuni	14	9	5	53	48	5
**EcN + *C. jejuni***	32	21	11	59	51	8

Supplemental Table 2: Fold change of the tight junction genes expression in response to different treatment. The up-regulated and down-regulated genes were determined using a cutoff ± ≥ 1.5 or ≤ 1.5 and a $P \leq 0.05$. Red, up-regulated genes; Green, down-regulated genes; Gray, no change

**2h post-infection**				
**Genes**	**Gene product description**	**EcN**	**Cj**	**EcN+ Cj**
ACTN1	Actinin, alpha 1	2.1		
ACTN4	Actinin, alpha 4	1.8		
ARHGEF2	Rho/rac guanine nucleotide exchange factor (GEF) 2	3.0		1.9
ASH1L	Ash1 (absent, small, or homeotic)	1.5		
CASK	Calcium/calmodulin-dependent serine protein kinase	1.5		
CD99	CD99 molecule	1.7		
CDC42	Cell division cycle 42 (GTP binding protein)			1.7
CDK4	Cyclin-dependent kinase 4	-1.5		
CGN	Cingulin	3.6	2.0	2.2
CLDN3	Claudin 3	2.0		1.5
CLDN5	Claudin 5	1.5		1.9
CLDN6	Claudin 6		-2.3	-2.3
CLDN8	Claudin 8			-1.6
CLDN9	Claudin 9	1.7		2.4
CLDN10	Claudin 10			-2.5
CLDN11	Claudin 11	-4.5	-3.8	-6.6
CLDN15	Claudin 15	1.5	-1.7	1.6
CLDN18	Claudin 18			-1.5
CLDN19	Claudin 19	-1.7		-3.4
CSDA	Cold shock domain protein A	1.8		
CSNK2A1	Casein kinase 2, alpha 1 polypeptide	1.7		
CSNK2A2	Casein kinase 2, alpha prime polypeptide	2.2		
CTNNA2	Catenin (cadherin-associated protein), alpha 2			1.6
CTNNB1	Catenin (cadherin-associated protein), alpha 3	-1.7		
CTTN	Cortactin	1.5		
EPB41	Erythrocyte membrane protein band 4.1	1.6		
ESAM	Endothelial cell adhesion molecule	1.6		2.1
F11R	F11 receptor	1.8		1.6
GNAI1	polypeptide 1	1.5		
HCLS1	Hematopoietic cell-specific Lyn substrate 1		-4.2	-6.6
ICAM1	Intercellular adhesion molecule 1	3.3		1.9
IGSF5	Immunoglobulin superfamily, member 5	1.5	2.3	1.5
ILK	Integrin-linked kinase		1.5	
INADL	InaD-like	1.5		
JAM2	Junctional adhesion molecule 2	-1.9		
JAM3	Junctional adhesion molecule 3	-2.1		-2.4
LLGL2	Lethal giant larvae homolog 2	2.7	1.5	2.1
MAGI1	Membrane associated guanylate kinase	1.8		

2h post-infection continued

MARK2	MAP/microtubule affinity-regulating kinase 2	2.1		1.5
MLLT4	Myeloid/lymphoid or mixed-lineage leukemia	2.3		1.6
MPDZ	Multiple PDZ domain protein	1.5	-1.5	
MPP6	Membrane protein, palmitoylated 6	1.5		
PARD3	Par-3 partitioning defective 3 homolog			-1.5
PARD6B	Par-6 partitioning defective 6 homolog beta	-1.7		
PECAM1	Platelet/endothelial cell adhesion molecule			-1.7
PRKCZ	Protein kinase C, zeta	2.0	2.5	1.6
PTEN	Phosphatase and tensin homolog	1.8	1.6	
RAC1	Ras-related C3	1.8		
SPTAN1	Spectrin, alpha 1	1.7		1.5
SPTB	Spectrin	1.9	1.6	1.5
SYMPK	Symplekin	2.1		1.5
TJAP1	Tight junction associated protein 1 (peripheral)	1.8	2.1	1.9
ZO1	Zona occludens 1 (tight junction protein 1)			-1.5
ZO2	Zona occludens 2 (tight junction protein 2)	1.9		
ZO3	Zona occludens 3 (tight junction protein 3)	4.0	1.7	1.9

Supplemental Table 2: Fold change of the tight junction genes expression in response to different treatment. The up-regulated and down-regulated genes were determined using a cutoff ± ≥ 1.5 or ≤ 1.5 and a P ≤ 0.05. Red, up-regulated genes; Green, down-regulated genes; Gray, no change

24h post-infection

Genes	Gene product description	EcN	Cj	EcN+ Cj
ACTN1	Actinin, alpha 1	1.9	1.8	1.9
ACTN2	Actinin, alpha 2	42.5	-5.1	-1.6
ACTN3	Actinin, alpha 3	87.0	1.7	4.2
ACTN4	Actinin, alpha 4	2.2	2.0	3.5
ARHGEF2	Rho/rac guanine nucleotide exchange factor (GEF) 2		1.7	2.1
ASH1L	Calcium/calmodulin-dependent serine protein kinase	1.5	2.1	1.5
CD99	CD99 molecule	4.0		2.3
CGN	Cingulin	2.3	2.8	5.0
CLDN1	Claudin 1	-1.6		
CLDN2	Claudin 2	1.6	-2.4	1.6
CLDN3	Claudin 3	2.4		1.7
CLDN4	Claudin 4	1.6	-1.5	2.2
CLDN5	Claudin 5	34.7	6.8	2.5
CLDN6	Claudin 6	11.3	4.3	6.9
CLDN7	Claudin 7	1.5		-1.5
CLDN8	Claudin 8	33.8	12.5	
CLDN9	Claudin 9	4.5	2.3	5.4
CLDN10	Claudin 10	4.1	8.0	5.4
CLDN11	Claudin 11	3.6	-1.6	1.9
CLDN12	Claudin 12			-1.7
CLDN14	Claudin 14	101.0	27.3	32.7
CLDN15	Claudin 15	2.2	2.6	2.3
CLDN16	Claudin 16		2.4	3.7
CLDN18	Claudin 18		3.0	
CLDN19	Claudin 19	4.4	2.5	3.3
CRB1	Crumbs homolog 1	33.6		-8.5
CRB3	Crumbs homolog 3		2.9	2.5
CSDA	Cold shock domain protein A	1.6		2.0
CSNK2A2	Casein kinase 2, alpha prime polypeptide	2.7	1.7	2.3
CTNNA2	Catenin (cadherin-associated protein), alpha 2	18.0	1.6	1.8
CTNNA3	Catenin (cadherin-associated protein), alpha 3	11.6		-4.4
CTNNB1	Catenin (cadherin-associated protein), alpha 3	11.7		-1.9
CTTN	Cortactin	5.1	1.6	3.8
EPB41	Erythrocyte membrane protein band 4.1	2.0	3.3	1.7
ESAM	Endothelial cell adhesion molecule	2.8		2.4
F11R	F11 receptor		1.7	
GNAI1	polypeptide 1			1.5
HCLS1	Hematopoietic cell-specific Lyn substrate 1	7.4		
ICAM1	Intercellular adhesion molecule 1	5.9	8.2	9.8

24h post-infection continued

ICAM2	Intercellular adhesion molecule 2	8.2	2.6	2.1
IGSF5	Immunoglobulin superfamily, member 5	13.3	-1.7	-1.6
INADL	InaD-like		1.6	
JAM2	Junctional adhesion molecule 2	2.7	8.1	1.8
JAM3	Junctional adhesion molecule 3	2.8	2.6	3.6
LLGL1	Lethal giant larvae homolog 1	9.9		2.3
LLGL2	Lethal giant larvae homolog 2	7.6	1.5	2.7
MAGI1	Membrane associated guanylate kinase	4.5	2.9	2.1
MARK2	MAP/microtubule affinity-regulating kinase 2	7.0	5.0	6.6
MLLT4	Myeloid/lymphoid or mixed-lineage leukemia	2.3	2.2	3.6
MPDZ	Multiple PDZ domain protein	2.9	2.9	1.5
MPP6	Membrane protein, palmitoylated 6	1.5	2.2	2.1
OCLN	Occludin		4.0	2.5
PARD3	Par-3 partitioning defective 3 homolog			-1.7
PARD6A	Par-6 partitioning defective 6 homolog alpha	2.9		2.2
PARD6B	Par-6 partitioning defective 6 homolog beta	2.5	3.7	6.4
PECAM1	Platelet/endothelial cell adhesion molecule	17.8	4.1	6.7
PRKCI	Protein kinase C, iota		1.7	
PRKCZ	Protein kinase C, zeta	7.0	3.1	4.0
RHOA	Ras homolog gene family, member A			-1.5
SMURF1	SMAD specific E3 ubiquitin protein ligase 1	2.8	4.7	4.8
SPTA1	Spectrin, alpha 1 (elliptocytosis 2)	15.3		
SPTAN1	Spectrin, alpha 1	2.6	1.5	3.7
SPTB	Spectrin, beta	1.8	2.1	1.9
SYMPK	Symplekin	7.1	2.5	3.2
TJAP1	Tight junction associated protein 1 (peripheral)	4.0	1.6	3.8
ZO1	Zona occludens 1 (tight junction protein 1)		1.7	1.8
ZO2	Zona occludens 2 (tight junction protein 2)	1.6	1.8	2.0
ZO3	Zona occludens 3 (tight junction protein 3)	2.1	1.7	2.1
VAPA	vesicle-associated membrane protein-associated protein A		1.8	
ZAK	Sterile alpha motif and leucine zipper containing kinase AZK		1.9	
