# Supplementary material for: In Vitro Evaluation of the Impact of the Probiotic E. coli Nissle 1917 on Campylobacter jejuni’s Invasion and Intracellular Survival in Human Colonic Cells
Source: Front Microbiol. 2017 Aug 22;8:1588. doi: 10.3389/fmicb.2017.01588 (PMC5572226; doi:10.3389/fmicb.2017.01588)
Supplement: Supplementary file 2 [file Image_1.PDF]

Supplementary figure 1

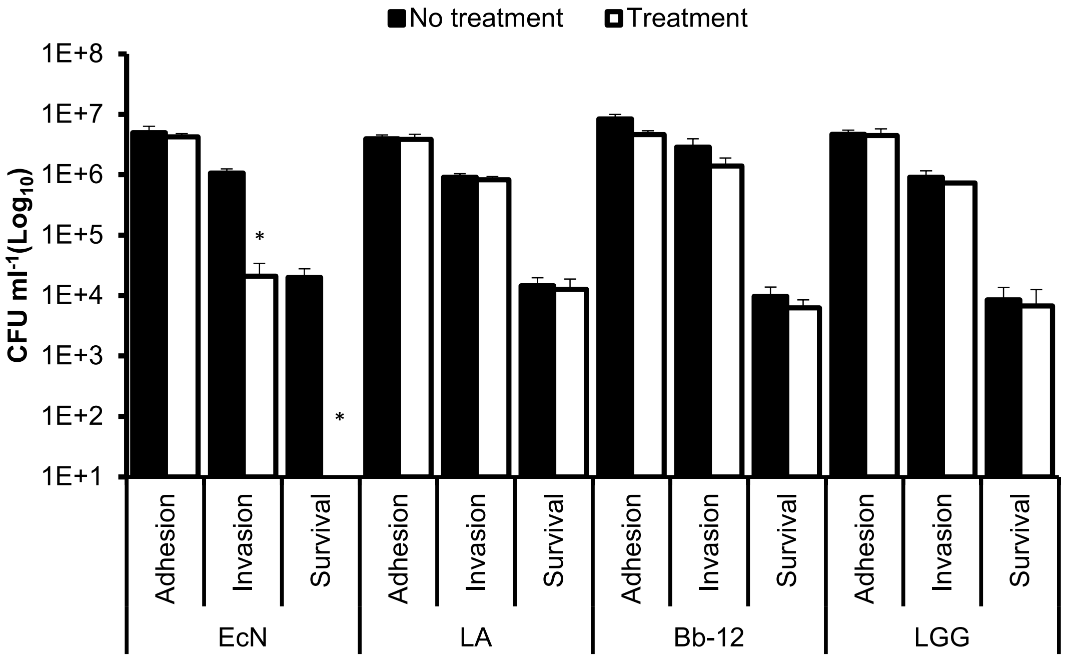

Supplemental Figure S1: *C. jejuni* adhesion to, invasion of and intracellular survival in Caco-2 cells (ATCC® HTB-37™) pretreated with EcN, *Lactobacillus acidophilus* NCFM (LA), *Bifidobacterium animalis subsp. Lactis* (Bb-12) and *Lactobacillus rhamnosus* GG (LGG) for 4 h. *indicates statistically significant differences ($P < 0.05$) in *C. jejuni* CFU numbers between probiotic-treated and non-treated Caco-2 cells. The experiments were repeated two times and samples were processed in 4 replicates in each experiment.
